# Computing edge version of metric dimension of certain chemical networks

**DOI:** 10.1038/s41598-024-62063-6

**Published:** 2024-05-26

**Authors:** Muhammad Umer Farooq, Muhammad Hussain, Ahmed Zubair Jan, Afraz Hussain Mjaeed, Mirwais Sediqma, Ayesha Amjad

**Affiliations:** 1 Department of Mathematics, COMSATS University Islamabad, Lahore Campus, Punjab, 53710 Pakistan; 2 Department of Mechanical Engineering Wroclaw, University of Science and Technology, Wrocław , Poland; 3 School of Energy and Power Engineering, Jiangsu University, Zhenjiang, 212013 China; 4 Department of Civil Engineering, University of Laghman, Lagham, Afghanistan; 5 Faculty of Transport and Aviation Engineering, Silesian University of Technology, Katowice, Poland

**Keywords:** Resolving set, Basis, Metric dimension, Edge metric dimension, Bakelite network, Backbone DNA network, Polythiophene, Concealed Non-Kekuléan Benzenoid Hydrocarbon, Engineering, Mathematics and computing

## Abstract

In the modern digital sphere, graph theory is a significant field of research that has a great deal of significance. It finds widespread application in computer science, robotic directions, and chemistry. Additionally, graph theory is used in robot network localization, computer network problems and the formation of various chemical structures for networks. Moreover, it finds uses in exploring diffusion mechanisms and scheduling aircraft as well. The present research project examines and concentrates on the edge version of metric dimension of the Concealed Non-Kekuléan Benzenoid Hydrocarbon, Polythiophene, Backbone DNA network and Bakelite networks. All the mentioned networks have constant edge metric dimension except Bakelite network, as demonstrated by the results. If we talk about the applications of these networks, Polythiophene are used to treat prion disorders. It is also capable of detecting metal ions. The concept of Bakelite, which finds applications in the jewelry, electrical, cookware, sports, and clock industries, had an impact on the invention of modern polymers. The functions of DNA include information encoding, replication, mutation, and recombination gene expression.

## Introduction

*M* is referred to as the generator of the metric space if each point in the metric space can be uniquely identified by its distances from the elements of *M*, where *M* is the set of points. There are various kinds of metric generators in graphs in the present day. According to its uses and popularity each one of them studied in applied and theoretical ways. However, many more perspectives remain unconsidered when describing a graph in these metric generators.

The ability of a graph to resolve problems, or to uniquely identify the positions of its vertices, is quantified by its metric dimension. Numerous fields have examined the concept of metric dimension, most notably molecular chemistry, social networks, and communication networks. The metric dimension of several graph models has been investigated, including entire graphs, grids, and cycles. In order to comprehend this concept, it is essential to first acquire an understanding of the concept of distance in connected graphs. In a connected graph *G*, the distance between two vertices *a* and *b* is interpreted as the length of the shortest path between them and is symbolized by *d*(*a*, *b*). $``Dim(G)''$ indicates the metric dimension of a graph *G*. Let $a\in V$ represents a vertex and let $e = bc\in E$ represents an edge in a connected graph $G=(V, E)$. Then $d_G(e, a) = {min\{d_G(b, a), d_G(c, a)}\}$ is the distance between the vertex *a* and the edge *e*. A vertex $c\in V$ distinguishes two edges $e_1, e_2\in E$ if $d_G(c, e_1)\ne d_G(c, e_2)$. If there is a vertex in set *M* that distinguished each of the two edges in a connected graph *G*, then that set of vertices is an edge metric generator for *G*. The edge metric dimension, *edim*(*G*), is the minimum cardinality of an edge metric generator for *G*.

In 1976, Harary and Melter^1^ proposed both the concept of a graph’s metric dimension as well as its computing methodology. Additionally, they proved that a graph’s diameter and its maximum degree are equal to the values of its metric dimension. The metric dimension of many types of graphs like trees, cycles, and complete graphs is investigated by Melter and Harary in 1984^2^. Furthermore, in accordance with the size and order of the graphs, they established bounds on their metric dimensions. Chartrand and Harary provided a review of the numerous discoveries and applications of the metric dimension of a graph in 1985^3^. Resolving sets were first proposed by Slater^4^ in 1988, who also showed that the metric dimension of a graph is equal to the minimal cardinality of a resolving set. Any collection of vertices in a graph *G* that ensures that each distance vector between those vertices and each of the other vertices in *G* is unique is called a resolving set. Oellermann and Pfaff’s 1993 review^5^ provided a thorough and in-depth summary of the numerous discoveries and uses of the metric dimension of a graph. The notion of k-metric dimension, which extends the metric dimension to k-resolving sets, was first presented by Khuller et al.^6^ in 1996. Pal and Das examined the metric dimension of disconnected graphs and offered a technique for calculating it in a paper published in 2000^7^. Nithya and Simon^8^ published a study in 2019 discussing the metric dimension of several chemical graphs, such as the Star of oxide network. They additionally put limitations on these graphs’ metric dimension in terms of their size and order.

These are simply just a few examples of the numerous research and articles that have been written about the metric dimension and its uses still it will provide you with an idea of the scope of this field’s work^9–11^.

In^12^, it has been claimed and then shown that the only graph with a metric dimension of 1 is a path graph. For $n\ge 3$, the cycle graph’s metric dimension is 2. Applications like space navigation and chemistry benefit greatly from this idea. When it comes to space routing, for instance, the objective is to assign the fewest robots possible to certain vertices so that they can visit each one precisely once. The idea of metric dimension can be used to solve this issue.

Numerous chemical compounds with identical chemical equations but distinct chemical structures can be found in chemistry. Chemists must choose the molecule that most effectively conveys its key chemical and physical characteristics. They need a scientific labeling system that assigns distinct labels to certain chemicals in order to do this. For scientists working on drug development, the numerical representation of distinct chemical compounds is essential. Chemical compounds can be represented using graphs, where atoms are represented by vertices and bond types by edges^13,14^. The publications^14–16^ cover theoretical explanations of graphs and their applications.

The most well-known area of graph theory concerned with graph distances has the name metric dimension. Edge version of metric dimension of the Concealed Non-Kekuléan Benzenoid Hydrocarbon, Polythiophene, Backbone DNA Network and Bakelite networks would be calculated after obtaining some insight from the most recent study on resolving characteristics of graphs^9–11,17–19^. Recently an outstanding work on metric dimension of line graph is published by Muhammad Umer in^20^. It has been proven in^21^ that the edge resolvability of $D'_n$ is bounded, while of $f_{n \times 3}$ and $D_n^t$ is unbounded.

### Definition 1

The term “**basis**” refers to a resolving set for a graph *G* that has the least number of vertices.^15^.

### Definition 2

Within a connected graph, its **Eccentricity**^22^ is the maximum distance between two vertices.

### Definition 3

The family of connected graphs with **Constant Edge metric dimension** is defined as one in which every graph in the family has the same metric dimension^23^.

## Concealed Non-kekuléan Benzenoid Hydrocarbon $(CNBH_{(m)})$

The hydrocarbons that are classified as benzenoid hydrocarbons are those that have six members and are classified as such in the chemical world. Burning natural resources in an inefficient manner results in the production of benzenoid hydrocarbons. Occasionally referred to as pitch or tar, the substance that is produced as a result of this incomplete combustion is characterized by its dark hue. The material used in this process and the environmental conditions ssuch as temperature under which this chemical reaction takes place influence the type and physics of the substance that is produced. Pitch and tar mixes are quite complicated; coal tar, for example, contains quite a few 100, 000 compounds. Two categories of hydrocarbon structures benzenoid are exist:

- Concealed non-kekuléan structure.
- Concealed kekuléan structure.

**Figure 1:**
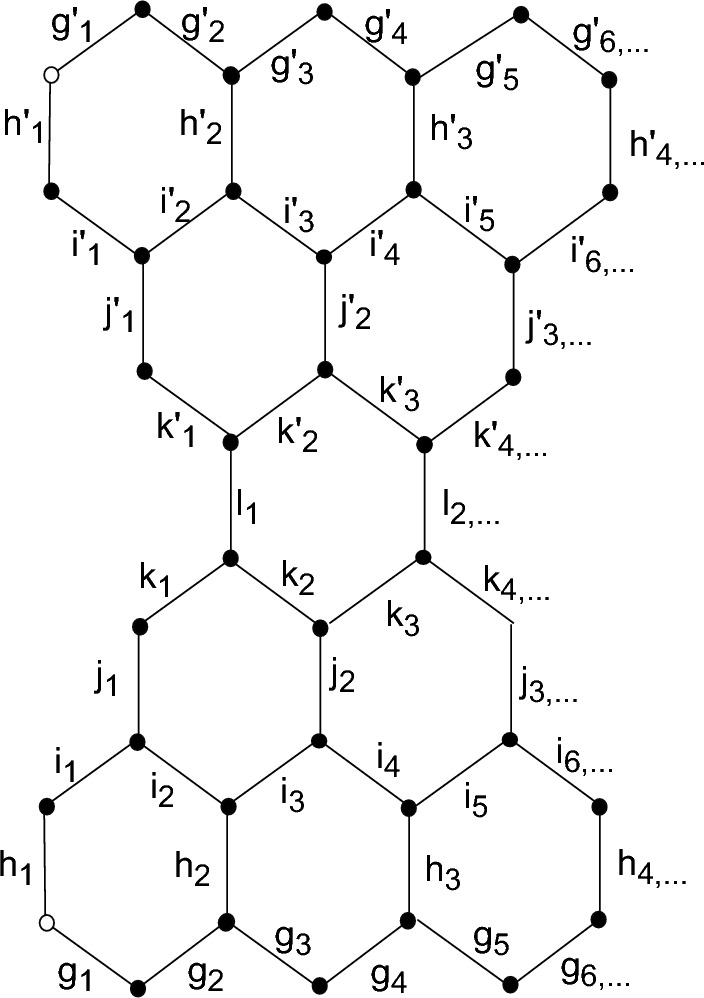
Labeling of CNBH for m=2.

It is considered a concealed non-kekuléan structure if the number of starred and unstarred nodes in the structure differs, meaning that it does not have any kekuléan nodes.. A structure is referred to as concealed kekuléan if it contains the same number or more number of starred and unstarred vertices. considering *n*, the number of edges connected in the central part of the graph, in the chain of concealed non-kekuléan benzenoid graph the total number of edges are $(17n+14)$. The equality of the number of starred and unstarred vertices is a essential as well as sufficient requirement for a benzenoid structure to be Kekuléan, as addressed by Gutman in^24^. Even while just two naphthalene compounds benzenoid hydrocarbons and anthracene have a substantial number of applications in the chemical industry, one may argue that pitches and tars have no direct relation for various mechanical and technological applications. The method by which over 65% of coal tar is transformed into phthalic anhydride is known as catalytic gas-phase oxidation. Additionally, the production of polymers make use of this product. The synthetic production of naphthalene sulfonic acid, p-naphthol, and other colors uses around 15% of naphthalene. There is a trace quantity of naphthalene in moth balls too. Figure 1 shows successive molecule of the non-kekuléan benzenoid hydrocarbon that is the subject of this study.

We can label molecules of concealed non-kekuléan benzenoid hydrocarbon as shown in the following Fig. 1.

### Theorem 4

*The graph* $G \cong CNBH_{(m)}$ *has edge edge metric dimension* 2. where $m=1,2,3,4,....$

### *Proof*

The general vertex set for $CNBH_{(m)}$ is, $V(CNBH_{(m)})=\{g_{z}: \ 1 \le z \le 2m+2\}\cup \{h_{z}:\ 1 \le z\le m+2\}\cup \{i_{z}:\ 1 \le z\le 2m+2\}\cup \{j_{z}:\ 1 \le z\le m+1\}\cup \{k_{z}:\ 1 \le z\le 2m\}\cup \{l_{z}:\ 1 \le z\le m\}\cup \{g'_{z}: \ 1 \le z \le 2m+2\}\cup \{h'_{z}:\ 1 \le z\le m+2\}\cup \{i'_{z}:\ 1 \le z\le 2m+2\}\cup \{j'_{z}:\ 1 \le z\le m+1\}\cup \{k'_{z}:\ 1 \le z\le 2m\}$

$$\begin{aligned} \lambda (g_z)= \left\{ \begin{array}{llll} z-1, 9 \quad &{} for \quad &{}z=1,2,3,4,5\\ z-1, z+4 \quad \quad &{} for \quad &{}z>5\\ \end{array} \right. \end{aligned}$$

$$\begin{aligned} \lambda (h_z)= \left\{ \begin{array}{llll} 2z-2,8 \quad \quad &{}for \quad \quad &{}z=1,2,3\\ 2z-2,2z+2 \quad \quad &{}for \quad \quad &{}z>3\\ \end{array} \right. \end{aligned}$$

$$\begin{aligned} \lambda (i_z)= \left\{ \begin{array}{llll} z, 7 \quad \quad &{}for \quad \quad &{}z=1,2,3,4\\ z, z+3 \quad \quad &{}for \quad \quad &{}z\ge 5\\ \end{array} \right. \end{aligned}$$

$$\begin{aligned} \lambda (j_z)= \left\{ \begin{array}{llll} 2z, 6 &{}for \quad \quad &{} z=1,2\\ 2z, 2z+2 \quad \quad &{}for \quad \quad &{}z>2\\ \end{array} \right. \end{aligned}$$

$$\begin{aligned} \lambda (k_z)= \left\{ \begin{array}{llll} z+2,5 \quad \quad &{}for &{} z=1,2\\ z+2,z+3 \quad \quad &{} for \quad \quad &{} z>2\\ \end{array} \right. \end{aligned}$$

$$\begin{aligned} \lambda (l_z)= \left\{ \begin{array}{llll} 2z+2, 2z+2 &{}for &{} z=1,2,3,...,m\\ \end{array} \right. \end{aligned}$$

$$\begin{aligned} \lambda (g'_z)= \left\{ \begin{array}{llll} 9, z-1 \quad \quad &{} for &{}z=1,2,3,4,5\\ z+4, z-1 \quad \quad &{} for \quad \quad &{} z>5\\ \end{array} \right. \end{aligned}$$

$$\begin{aligned} \lambda (h'_z)= \left\{ \begin{array}{llll} 8, 2z-2 \quad \quad &{}for \quad \quad &{} z=1,2,3\\ 2z+2, 2z-2 \quad \quad &{}for \quad \quad &{}z>3\\ \end{array} \right. \end{aligned}$$

$$\begin{aligned} \lambda (i'_z)= \left\{ \begin{array}{llll} 7, z \quad \quad &{}for \quad \quad &{} z=1,2,3,4\\ z+3, z \quad \quad &{}for \quad \quad &{}z\ge 5\\ \end{array} \right. \end{aligned}$$

$$\begin{aligned} \lambda (j'_z)= \left\{ \begin{array}{llll} 6, 2z &{}for \quad \quad &{} z=1,2\\ 2z+2, 2z \quad \quad &{}for \quad \quad &{}z>2\\ \end{array} \right. \end{aligned}$$

$$\begin{aligned} \lambda (k'_z)= \left\{ \begin{array}{llll} 5, z+2 \quad \quad &{} for &{} z=1,2\\ z+3, z+2 \quad \quad &{}for \quad \quad &{}z>2\\ \end{array} \right. \end{aligned}$$

$CNBH_{(m)}$ has edge metric dimension 2. $\square$

**Figure 2:**
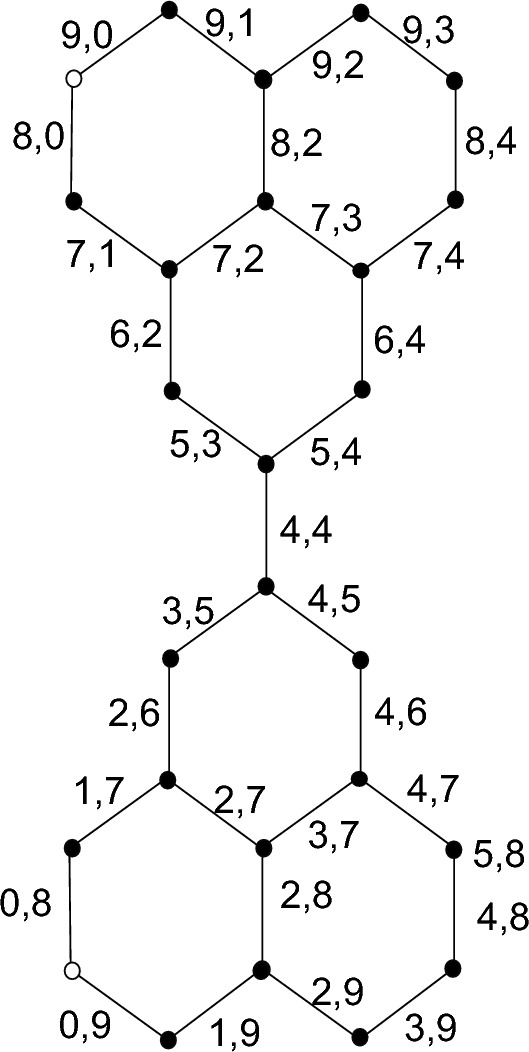
Edge edge metric dimension of CNBH for $m=1$.

Since the only graph with edge metric dimension 1 is the path graph^25,26^ and our above graph is not a path graph. On the behalf of this reason we can easily say that edge metric dimension of $CNBH_{(m)}$ is not 1. If $m=1$ then the pattern of edge metric dimension of $CNBH_{(1)}$ is shown in Fig. 2.

## Polythiophene network $(PT_{(m)})$

This section focuses into the chemical foundation of the polythiophene network $(PT_{(m)})$ and discusses some of the uses of polythiophene too. Subsequently, it was demonstrated by theorem that the polythiophene network $(PT_{(m)})$ has a constant edge metric dimension.

Polymerized thiophene is referred to as Polythiophene. It is a ring composed of five components, one of which is a heteroatom connected to their benzo and the other carbocyclic. It has an order of 5*n* and a size of $6m-1$. The discovery of *Pi*-conjugated conducting polymers has significantly influenced the field of organic electronics. When polythiophene is oxidized, it becomes a conductor. Because of their ability to switch between conducting and semiconducting states, these polymers may be integrated into electrical and optical devices. Polythiophene is one of the most researched polymers mathematically and experimentally among all *Pi*-conjugated polymers. Polythiophene is being studied experimentally because of its applications in electrical devices such as biosensors, hydrogen storage, water purification devices, and light-emitting diodes.In recognition of their research and development of polythiophenes and related conductive organic polymers, Alan J. Heeger, Alan MacDiarmid, and Hideki Shirakawa were granted the Chemistry Nobel Prize. Polythiophenes (PTs) are used to treat prion disorders. It is also capable of detecting metal ions.

**Figure 3:**
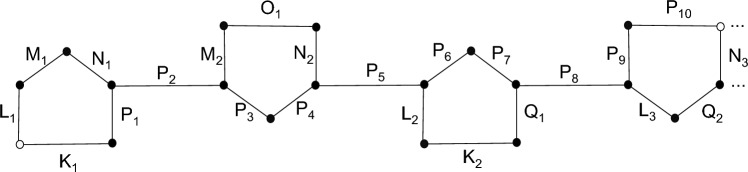
Labeling pattern of $(PT_{(m)})$.

Pattern of labeling of polythiophene network $(PT_{(m)})$ is shown in following Fig. 3.

### Theorem 5

*The graph* $G \cong (PT_{(m)})$ *has edge metric dimension* 2. where $m=1,2,3,4,.....$

### *Proof*

The general vertex set for $(PT_{(m)})$ is

$$\forall \ m=1,2,3,4...$$

$$V(PT_{(m)})=\{P_{z}: \ 1 \le z \le 3m-2\}$$

$$\forall \ m=1,3,5...$$

$$V(PT_{(m)})=\{K_{z}: \ 1 \le z \le \frac{m+1}{2}\} \cup \{L_{z}: \ 1 \le z \le \frac{m+1}{2}\}\cup \{M_{z}: \ 1 \le z \le \frac{m+1}{2}\}\cup \{N_{z}: \ 1 \le z \le \frac{m+1}{2}\}\cup \{O_{z}: \ 1 \le z\le \frac{m-1}{2}\}\cup \{Q_{z}: \ 1 \le z \le \frac{m-1}{2}\} \forall \ m=2,4,6...$$ $$V(PT_{(m)})=\{K_{z}: \ 1 \le z \le \frac{m}{2}\} \cup \{L_{z}: \ 1 \le z \le \frac{m+2}{2}\}\cup \{M_{z}: \ 1 \le z \le \frac{m}{2}\}\cup \{N_{z}: \ 1 \le z \le \frac{m+2}{2}\}\cup \{O_{z}: \ 1 \le z\le \frac{m}{2}\}\cup \{Q_{z}: \ 1 \le z \le \frac{m}{2}\}$$

$$\begin{aligned} \lambda (K_z)= \left\{ \begin{array}{lllll} 0,3m-6z+4 \quad \quad &{} for \quad \quad &{} z=1 \quad \quad &{} m=1,2,3,...\\ 6z-5,3m-6z+4 \quad \quad &{} for \quad \quad &{} 1<z\le \dfrac{m}{2} \quad \quad &{} m=2,4,6,...\\ 6z+1,3m-6z+4 \quad \quad &{} for \quad \quad &{} 1<z\le \dfrac{m}{2} \quad \quad &{} m=1,3,5,...\\ \end{array} \right. \end{aligned}$$

Here we define new terms $A_1$ and $A_2$ as follows:

$$A_1$$
: First entry of vertex.
$$A_2$$
: Second entry of vertex.

$$\begin{aligned} \lambda (L_z)= \left\{ \begin{array}{lllll} A_1, A_2 \quad \quad where\\ A_1: 0 \quad \quad &{} for \quad \quad &{} z=1 \quad \quad &{} m=1,2\\ A_1: 6z-6 \quad \quad &{} for \quad \quad &{} 1<z< \dfrac{m+2}{2} \quad \quad &{} m=2,4,6,...\\ A_1: 3m-3 \quad \quad &{}for \quad \quad &{} z= \dfrac{m+2}{2} \quad \quad &{} m=2,4,6,... \\ A_1:3m-3 \quad \quad &{}for \quad \quad &{} z= \dfrac{m+1}{2} \quad \quad &{} m=1,3,5,7...\\ A_1:6z-6 \quad \quad &{}for \quad \quad &{} 1<z<\dfrac{m+1}{2} \quad \quad &{} m=1,3,5,7...\\ A_2:2 \quad \quad &{}for \quad \quad &{} z= \dfrac{m+2}{2} \quad \quad &{} m=2,4,6,...\\ A_2:2 \quad \quad &{}for \quad \quad &{} z= \dfrac{m+1}{2} \quad \quad &{} m=1,3,5,7...\\ A_2:3m-6z+5 \quad \quad &{}for \quad \quad &{} z= 1 \quad \quad &{} m=1,2,3,4,...\\ A_2:3m-6z+5 \quad \quad &{}for \quad \quad &{} 1<z< \dfrac{m+1}{2} \quad \quad &{} m=1,3,5,7...\\ A_2:3m-6z+5 \quad \quad &{}for \quad \quad &{} 1<z< \dfrac{m+2}{2} \quad \quad &{} m=2,4,6,...\\ \end{array} \right. \end{aligned}$$

$$\begin{aligned} \lambda (M_z)= \left\{ \begin{array}{lllll} A_1, A_2 \quad \quad where\\ A_1: 1 &{} for \quad \quad &{} z=1 \quad \quad &{}m=1,2,3,...\\ A_1: 6z-9 \quad \quad &{}for \quad \quad &{} 1<z\le \dfrac{m}{2} \quad \quad &{} m=2,4,6,...\\ A_1: 6z-9 \quad \quad &{}for \quad \quad &{} 1<z\le \dfrac{m+1}{2} \quad \quad &{} m=1,3,5,... \\ A_2:3m-2 \quad \quad &{}for \quad \quad &{} z=1 \quad \quad &{} m=1,2,3,4,...\\ A_2:3m-6z+8 \quad \quad &{}for \quad \quad &{} 1<z<\dfrac{m}{2} \quad \quad &{} m=2,4,6,...\\ A_2:3z-1 \quad \quad &{}for \quad \quad &{} z= \dfrac{m+1}{2} \quad \quad &{} m=3,5,7...\\ A_2:3m-6z+5 \quad \quad &{}for \quad \quad &{} 1<z< \dfrac{m+1}{2} \quad \quad &{} m=3,5,7,...\\ \end{array} \right. \end{aligned}$$

$$\begin{aligned} \lambda (N_z)= \left\{ \begin{array}{lllll} A_1, A_2 \quad \quad where\\ A_1: 2 &{} for \quad \quad &{} z=1 \quad \quad &{} m=1,2,3,...\\ A_1: 6z-7 \quad \quad &{}for \quad \quad &{} 1<z\le \dfrac{m+2}{2} \quad \quad &{} m=2,4,6,...\\ A_1: 6z-7 \quad \quad &{}for \quad \quad &{} 1<z\le \dfrac{m+2}{2} \quad \quad &{} m=1,3,5,... \\ A_2:3m-3 \quad \quad &{}for \quad \quad &{} z= 1 \quad \quad &{} m=1,2,3,...\\ A_2:0 &{} for \quad \quad &{} z=\dfrac{m+2}{2} \quad \quad &{} m=2,4,6,...\\ A_2:3m-6z+6 \quad \quad &{}for \quad \quad &{} 1<z<\frac{m+2}{2} \quad \quad &{} m=4,6,8,...\\ A_2:3z-3 &{} for \quad \quad &{} z= \dfrac{m+1}{2} \quad \quad &{} m=3,5,7...\\ A_2:3m-6z+3 \quad \quad &{} for \quad \quad &{} 1<z< \dfrac{m+1}{2} \quad \quad &{} m=5,7,9,...\\ \end{array} \right. \end{aligned}$$

$$\begin{aligned} \lambda (O_z)= \left\{ \begin{array}{lllll} A_1, A_2 \quad \quad where\\ A_1: 6z-2 &{} for \quad \quad &{} z=2,4,6,... \quad \quad &{} m=1,2,3,...\\ A_2: 0 &{} for \quad \quad &{} z= \dfrac{m}{2} \quad \quad &{} m=4,6,8,...\\ A_2: 3m-6z+1 \quad \quad &{}for \quad \quad &{} 1 \le z \le \dfrac{m-1}{2} \quad \quad &{} m=1,3,5,... \\ \end{array} \right. \end{aligned}$$

$$\begin{aligned} \lambda (P_z)= \left\{ \begin{array}{lllll} A_1, A_2 \quad \quad where\\ A_1: z &{} for \quad \quad &{} 1 \le z \le 3m-2 \quad \quad &{} m=1,2,3,...\\ A_2: 3m-z-2 \quad \quad &{}for \quad \quad &{} 1 \le z \le 3m-2 \quad \quad &{} m=1,2,3,...\\ \end{array} \right. \end{aligned}$$

$$\begin{aligned}\lambda (Q_z)= \left\{ \begin{array}{lllll} A_1, A_2 \quad \quad where\\ A_1: 6z-2 &{} for \quad \quad &{} 1 \le z \le \dfrac{m}{2} \quad \quad &{} m=2\\ A_1: 6z+2 \quad \quad &{}for \quad \quad &{} 1\le<z< \dfrac{m}{2} \quad \quad &{} m=4,6,8,...\\ A_1: 6z+4 \quad \quad &{}for \quad \quad &{} z= \dfrac{m}{2} \quad \quad &{} m=4,6,8,... \\ A_1:6z+2 \quad \quad &{}for \quad \quad &{} 1 \le z \le \dfrac{m-1}{2} \quad \quad &{} m=3,5,7,...\\ A_2:1 for \quad \quad &{} 1 \le z \le \dfrac{m}{2} \quad \quad &{} m=2\\ A_2:3m-6z-3 \quad \quad &{}for \quad \quad &{} 1 \le z < \dfrac{m}{2} \quad \quad &{} m=4,6,8,...\\ A_2:3m-6z+1 &{} for \quad \quad &{} z= \dfrac{m}{2} \quad \quad &{} m=4,6,8,...\\ A_2:3m-6z-3 \quad \quad &{}for \quad \quad &{} 1 \le z \le \dfrac{m-1}{2} \quad \quad &{} m=3,5,7,...\\ \end{array} \right. \end{aligned}$$

$PT_{(m)}$ has edge metric dimension 2 which means it has a resolving set *B* with two vertices. Since $PT_{(m)}$ is not a path and hence has no edge metric dimension of 1, only path graphs have edge metric dimensions of one^25^. $\square$

**Figure 4:**
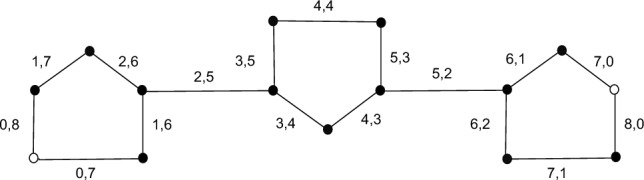
Edge metric dimension of $(PT_{(m)})$.

The diagram in Fig. 4 shows the edge edge metric dimension of polythiophene network $PT_m$.

## Backbone DNA network $BB_{DNA_{(m)}}$

This section provides an explanation of the chemical backdrop of the backbone DNA network $BB_DNA_(m)$, and the topology of the backbone DNA network is elaborated using a Figure. The backbone DNA network $BB_DNA_(m)$ is then shown to have a constant edge metric dimension.

**Figure 5:**
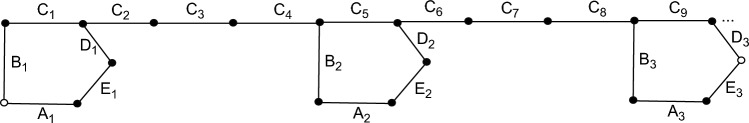
Labeling for $$BB_{DNA_{(3)}}$$.

Pattern of labeling of backbone DNA network $BB_{DNA_{(m)}}$ is shown in following Fig. 5.

DNA has two fundamental structural domains. The two components of DNA are its bases and its backbone. DNA stands for deoxyribonucleic acid in its whole form. The building blocks of DNA include alternating phosphate units, nitrogenous bases such as adenine, cytosine, guanine, and thymine, and deoxyribose, a pentose or five-carbon sugar. A DNA molecule is formed when several nucleotides are joined by phosphodiester linkages. DNA does not contain six-carbon or hexose carbohydrates. The backbone DNA network has a size of $8m-3$ and an order of 7*m*. DNA has a negative charge, and the proteins that eukaryotes call histones surrounding DNA molecules contain a large amount of positively charged amino acids.Histones and DNA become highly attracted to one other as a result. The functions of DNA include information encoding, replication, mutation, and recombination gene expression.

### Theorem 6

*The graph* $G \cong (BB_{DNA(m)})$ *has edge metric dimension* 2. where $m=1,2,3,4,....$

**Proof:**   By labeling all the vertices of backbone DNA network $(BB_{DNA(m)})$ we get the following general vertex set for $(BB_{DNA(m)})$,

$$V(BB_{DNA(m)})=\{A_{u}: \ 1 \le x \le m\}\cup \{B_{u}: \ 1 \le x \le m\}\cup \{C_{u}: \ 1 \le x \le 4m-3\}\cup \{D_{u}: \ 1 \le x \le m\}\cup \{E_{u}: \ 1 \le x \le m\}$$ where $$m=1,2,3...$$

Here we define new terms $X_1$ and $X_2$ as follows:

$$X_1$$
: First entry of vertex.
$$X_2$$
: Second entry of vertex.

$$\begin{aligned} \lambda (A_u)= \left\{ \begin{array}{lllll} X_1, X_2 \quad \quad where\\ X_1: 0 \quad \quad &{} for \quad \quad &{} u=1 \quad \quad &{} m=1,2,3,...\\ X_1: 4u-2 \quad \quad &{}for \quad \quad &{} 1<u \le m \quad \quad &{} m=1,2,3,...\\ X_2: 1 &{} for \quad \quad &{} u= m \quad \quad &{} m=1,2,3,... \\ X_2:4m-4u+3 \quad \quad &{}for \quad \quad &{} 1 \le u< m \quad \quad &{} m=1,2,3,...\\ \end{array} \right. \end{aligned}$$

$$\begin{aligned} \lambda (B_u)= \left\{ \begin{array}{lllll} X_1, X_2 \quad \quad where\\ X_1: 0 for \quad \quad &{} u=1 \quad \quad m=1,2,3,...\\ X_1: 4u-3 \quad \quad &{}for \quad \quad &{} 1<u \le m \quad \quad &{} m=1,2,3,...\\ X_2: 4m-4u+2 &{} for \quad \quad &{} u= 1 &{} m=1,2,3,... \\ X_2:4m-4u+2 \quad \quad &{}for \quad \quad &{} 1 < u \le m \quad \quad &{} m=1,2,3,...\\ \end{array} \right. \end{aligned}$$

$$\begin{aligned} \lambda (C_u)= \left\{ \begin{array}{lllll} X_1, X_2 \quad \quad where\\ X_1: u &{} for \quad \quad &{} 1 \le u \le 4m-3 \quad \quad &{} m=1,2,3,...\\ X_2: 4m-u-2 \quad \quad &{} for \quad \quad &{} 1 \le u \le 4m-3 \quad \quad &{} m=1,2,3,...\ \end{array} \right. \end{aligned}$$

$$\begin{aligned} \lambda (D_u)= \left\{ \begin{array}{lllll} X_1, X_2 \quad \quad where\\ X_1: 2 &{} for \quad \quad &{} u=1 \quad \quad m=1,2,3,...\\ X_1: 4u-3 \quad \quad &{}for \quad \quad &{} 1<u \le m \quad \quad &{} m=1,2,3,...\\ X_2: 0 &{} for \quad \quad &{} u= m &{} m=1,2,3,... \\ X_2:4m-4u+1 \quad \quad &{}for \quad \quad &{} 1 \le u< m \quad \quad &{} m=1,2,3,...\\ \end{array} \right. \end{aligned}$$

$$\begin{aligned}\lambda (E_u)= \left\{ \begin{array}{lllll} X_1, X_2 \quad \quad where\\ X_1: 1 &{} for \quad \quad &{} u=1 \quad \quad &{} m=1,2,3,...\\ X_1: 4u-1 \quad \quad &{} for \quad \quad &{} 1<u \le m \quad \quad &{} m=1,2,3,...\\ X_2: 0 for \quad \quad &{} u= m &{} m=1,2,3,... \\ X_2:4m-4u+2 \quad \quad &{}for \quad \quad &{} 1 \le u< m \quad \quad &{} m=1,2,3,...\\ \end{array} \right. \end{aligned}$$

**Figure 6:**
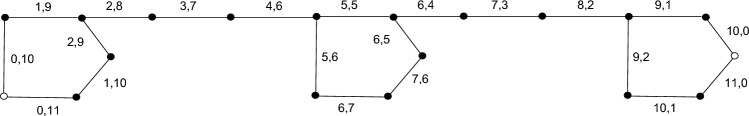
Edge edge metric dimension of $BB_{DNA_{(3)}}$.

Edge metric dimension is 2 for $BB_{DNA(m)}$. which means it has a resolving set with two vertices. Since $BB_{DNA(m)}$ is not a path and hence has no edge metric dimension of 1, only path graphs have edge metric dimensions of one^25^. The following Fig. 6 illustrates how we can determine the edge metric dimension of $BB_{DNA_{(m)}}$.

## Bakelite network $(B(n\times m))$

The Bakelite network $(B(n\times m))$’s chemical background is covered in this section. Then, as far as the constant edge metric dimension is concerned, we demonstrated by using a variety of theorems that the Bakelite network does not have it.

In 1907, phenol-formaldehyde resin also known as phenolic resin under chemical name bakelite, was created . The modern plastics industry had its start on this day. Upon applying a patent for a phenol-formaldehyde thermoset, American scientist Leo Hendrik Baekeland, a Belgian native, was recognized as the creator of Bakelite. Commercialization of the first entirely synthetic polymers was achieved by phenol-formaldehyde polymers, popularly referred to as plastics.The application of thermosetting polymers as adhesives covers about half of their current manufacturing.

This chemical substance named Bakelite finds extensive uses in numerous domains of human life. Among Bakelite’s many important applications some are:

- Within the electrical sector, it is employed in the production of non-conductive components for a wide range of electrical appliances, including light bulbs, supports, radios, phones, sockets, bases for electron tubes, and other silicon products.
- This kind of plastic is also used to make a lot of kitchenware items, like frying pans and unique kinds of spoon.
- It is used in the sports sector to manufacture billiard balls, chess sets, poker chips and various other game pieces.
- Playthings, clocks, and jewelry items are frequently made with it.
- For use in industrial and automotive components, its high resistivity to heat and electricity makes it a perfect material.

The generic Bakelite network, represented by $B(n\times m)$, consists of n rows and m columns, its development is credited with laying the groundwork for the current plastics industry.

### Theorem 7

*G* has edge metric dimension 2 for the set $G \cong B(1\times m)$ where *m* is a variable.

### *Proof*

One can generalize the vertex set for $B_{n\times m}$ as,

$$V(B(n\times m))=\{c_{y}^{z}: \ 1 \le y \le m, 1\le z\le m\}\cup \{d_{y}^{z}:\ 1 \le y\le m, 1\le z\le m\}\cup \{e_{y}^{z}:\ 1 \le y\le m, 1\le z\le 4m\}\cup \{i_{y}^{z}:\ 1 \le y\le m, 1\le z\le 4m\}\cup \{c`_{y}^{z}: \ 1 \le y \le m, 1\le z\le m\}\cup \{d`_{y}^{z}:\ 1 \le y\le m, 1\le z\le m\}\cup \{g_{y}^{z}: \ 1 \le y \le m, 1\le z\le m\}\cup \{h_{y}^{z}:\ 1 \le y\le m, 1\le z\le m\}\cup \{g`_{y}^{z}: \ 1 \le y \le m, 1\le z\le m\}\cup \{h`_{y}^{z}:\ 1 \le y\le m, 1\le z\le m\}\cup \{f_{y}^{z}:\ 1 \le y\le m, 1\le z\le 2m-2\}\cup \{f`_{y}^{z}:\ 1 \le y\le m, 1\le z\le m\}$$ where $$m=1,2,3,...$$

**Figure 7:**
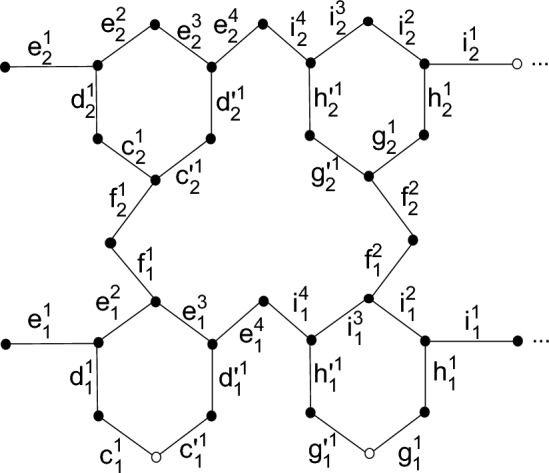
Labeling for $$BN_{2\times 1}$$.

We can label molecules of Bakelite network as shown in Fig. 7.

$$\begin{aligned} \lambda (c_{1}^{z})= \left\{ \begin{array}{lllll} 0,8m-4z+2 \quad \quad &{} for \quad \quad &{} z=1 \quad \quad &{} m=1,2,3,...\\ 4z-3, 8m-4z+2 \quad \quad &{} for \quad \quad &{} 1<z \le m \quad \quad &{} m=1,2,3,...\\ \end{array} \right. \end{aligned}$$

$$\begin{aligned} \lambda (g_{1}^{z})= \left\{ \begin{array}{lllll} 8m-4z+2,0 \quad \quad &{} for \quad \quad &{} z=1 \quad \quad &{} m=1,2,3,...\\ 8m-4z+2, 4z-3 \quad \quad &{}for \quad \quad &{} 1<z \le m \quad \quad &{} m=1,2,3,...\\ \end{array} \right. \end{aligned}$$

$$\begin{aligned} \lambda (c`_{1}^{z})= \left\{ \begin{array}{lllll} 0,8m-4z+1 \quad \quad &{} for \quad \quad &{} z=1 \quad \quad &{} m=1,2,3,...\\ 4z-2, 8m-4z+2 \quad \quad &{}for \quad \quad &{} 1<z \le m \quad \quad &{} m=1,2,3,...\\ \end{array} \right. \end{aligned}$$

$$\begin{aligned} \lambda (g`_{1}^{z})= \left\{ \begin{array}{lllll} 8m-4z+1,0 &{} for \quad \quad &{} z=1 \quad \quad &{} m=1,2,3,...\\ 8m-4z+1,4z-2 \quad \quad &{}for \quad \quad &{} 1<z \le m \quad \quad &{} m=1,2,3,...\\ \end{array} \right. \end{aligned}$$

$$\begin{aligned} \lambda (d_{1}^{z})= \left\{ \begin{array}{lllll} 1,8m-4z+2 &{} for \quad \quad &{} z=1 \quad \quad &{} m=1,2,3,...\\ 4z-4, 8m-4z+2 \quad \quad &{}for \quad \quad &{} 1<z \le m \quad \quad &{} m=1,2,3,...\\ \end{array} \right. \end{aligned}$$

$$\begin{aligned} \lambda (d`_{1}^{z})= \left\{ \begin{array}{lllll} 1,8m-4z &{} for \quad \quad &{} z=1 \quad \quad &{} m=1,2,3,...\\ 4z-2, 8m-4z \quad \quad &{}for \quad \quad &{} 1<z \le m \quad \quad &{} m=1,2,3,...\\ \end{array} \right. \end{aligned}$$

$$\begin{aligned} \lambda (h_{1}^{z})= \left\{ \begin{array}{lllll} 8m-4z+2, 1 &{} for \quad \quad &{} z=1 \quad \quad &{} m=1,2,3,...\\ 8m-4z+2,4z-4 \quad \quad &{}for \quad \quad &{} 1<z \le m \quad \quad &{} m=1,2,3,...\\ \end{array} \right. \end{aligned}$$

$$\begin{aligned} \lambda (h`_{1}^{z})= \left\{ \begin{array}{lllll} 8m-4z, 1 \quad \quad &{} for \quad \quad &{} z=1 \quad \quad &{} m=1,2,3,...\\ 8m-4z, 4z-2 \quad \quad &{}for \quad \quad &{} 1<z \le m \quad \quad &{} m=1,2,3,...\\ \end{array} \right. \end{aligned}$$

$$\begin{aligned} \lambda (e_{1}^{z})= \left\{ \begin{array}{lllll} 2,8m-z-1 \quad \quad &{} for \quad \quad &{} z=1,2,3 \quad \quad &{} m=1,2,3,...\\ m-2, 8m-z-1 \quad \quad &{}for \quad \quad v3<z \le m \quad \quad &{} m=1,2,3,...\\ \end{array} \right. \end{aligned}$$

$$\begin{aligned} \lambda (i_{1}^{z})= \left\{ \begin{array}{lllll} 8m-z-1, 2 \quad \quad &{} for \quad \quad &{} z=1,2,3 \quad \quad &{} m=1,2,3,...\\ 8m-z-1, m-2 \quad \quad &{}for \quad \quad &{} 3<z \le m \quad \quad &{} m=1,2,3,...\\ \end{array} \right. \end{aligned}$$

**Figure 8:**
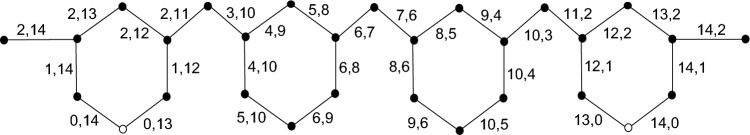
Edge edge metric dimension of $BN_{1\times 2}$.

Edge metric dimension is 2 for $B(1\times m)$. which means it has a resolving set with two vertices. Since $B(1\times m)$ is not a path and hence has no edge metric dimension of 1, only path graphs have edge metric dimensions of one^25^. The following Fig. 8 illustrates how we can determine the edge metric dimension of $B(1\times m)$. $\square$

### Theorem 8

*G* has edge metric dimension 3 for the set $G \cong B(2\times m)$ where $m=1,2$.

### *Proof*

In order to prove this theorem, we will show that graph *G* does not have a resolving set *H* with two vertices. On the contrary, we consider the case when graph *G* has edge metric dimension 2. First for the case $B(2\times 1)$, consider a resolving set $H=\{c_1^{1}{c'}_1^{1}, g_1^{1}{g'}_1^{1}\}$, then we have $r(c_2^{1}|H)=r(c`_2^{1}|H)$, and therefore, *H* is not a resolving set. Now lat us take another resolving set $H=\{c_1^{1}{c'}_1^{1}, c_2^{1}{c'}_2^{1}\}$ for $B(2\times 1)$ which results in $r(d_2^{1}|H)=r(d`_2^{1}|H)$, suggesting that *H* is not a resolving set for the graph. Suppose two more resolving sets $H=\{f_1^{1}{f}_2^{1}, f_1^{1}{f'}_1^{1}\}$ and $H=\{e_2^{1}{e}_2^{2}, f_1^{1}{f}_2^{1}\}$, then we will see more clashes as $r({d}_1^{1}|H)=r({e}_1^{1}|H)$ and $r({e}_1^{2}|H)=r({e}_1^{3}|H)$ respectively. Hence, it can be said clearly and with very ease that *H* is not a resolving set. Additionally, If we take resolving sets $H=\{e_2^{1}e_2^{2}, i_2^{1}{i}_2^{2}\}$ and $H=\{e_1^{1}{e}_1^{2}, f_2^{2}{f}_1^{2}\}$, then $r(e_1^{1}|H)=r(e_1^{2}|H)$ and $r(e_1^{1}|H)=r(e_1^{2}|H)$ is observable with very ease. This means that *H* is not a set that resolves. Similar to this, If we assume two last but not the least examples of resolving sets as $H=\{e_1^{1}e_1^{2},e_1^{4}{i}_1^{4}\}$ and $\{{c}_2^{1}c`_2^{1}, g_2^{1}{g`}_2^{1}\}$, then the results will be $r(c_2^{1}|H)=r(c`_2^{1}|H)$ and $r(e_1^{1}|H)=r(e_1^{2}|H)$ respectively. Hence, with no ambiguity we can claim that *H* is not a resolving set.

The particular case $B(2\times m)$ of Bakelite network for $m=1,2$, does not, in general, have a resolving set with two vertices. Its edge metric dimension is thus 3. $\square$

### Theorem 9

The graph $G \cong B(n\times m)$ has the following edge metric dimension:

$$\begin{aligned} Metric\ Dimension (G)= \left\{ \begin{array}{lllll} 4 \quad \quad &{} for \quad \quad &{} n=2\ and\ &{} m>2 \\ 4 \quad \quad &{}for \quad \quad &{} n\ge 3\ and\ &{} m>1 \end{array} \right. . \end{aligned}$$

### *Proof*

The method of contradiction will be employed in order to prove this theorem. The edge metric dimension of the graph *G* is 4, as we have specified in the above statement. On the other hand, we assume that the edge metric dimension of graph *G* is 3 and that earlier assumption is false. Let’s take an example of a resolving set in $B(2\times 3)$ as $H=\{c_1^{1}{c'}_{1}^{1},g_1^{1}{g'}_{1}^{1},i_2^{1}\}$, then we got the results as $r(i_2^{10}|H)=r(h_2^{3}|H)$. Therefore, *H* is not said to be a resolving set.

In the same way,consider two more resolving sets $H=\{c_1^{1}{c'}_{1}^{1},i_2^{1},g_1^{1}{g'}_{1}^{1}\}and \{c_1^{1}{c'}_{1}^{1},i_1^{10}{i}_{1}^{11},g_1^{1}{g'}_{1}^{1}\}$, then see following clashes $r(g_2^{1}|H)=r(g`_2^{1}|H)$, $r(g_2^{3}|H)=r(g`_2^{3}|H)$ respectively. which clearly indicates that here *H* is not a perfect resolving set.

The graph $G \cong B(2\times m)$, where $m=1,2$, does not, in general, have a resolving set with two vertices. Its edge metric dimension is thus 3. $\square$

### Theorem 10

*G* has edge metric dimension $n+1$ for the set $G \cong B(n\times 1)$ where *n* is a variable.

### *Proof*

Unit network of Bakelite $B(1\times 1)$ has edge metric dimension 2.

In the case of $B(2\times 1)$ the Edge metric dimension will be 3.

Edge metric dimension of Bakelite network for $B(3\times 1)$ is 4.

Continuing this process to the nth row, the edge metric dimension of Bakelite network for $B(n\times 1)$ is $n+1$. It proves that for $G \cong B(n\times 1)$ has edge metric dimension equal to $n+1$ where *n* varies. $\square$

## Concluding remarks

The concept of edge metric dimension is quite basic. But figuring out a graph’s precise edge metric dimension is an NP-complete problem. The Global Positioning System (GPS) and trilateration are strongly associated with the edge metric dimension. Localization of sources also makes advantage of it. The very high resistance of bakelite against heat and electricity makes it a valuable material for industrial and automotive components manufacturing. Due to its widespread usage in the production of non-conducting components for electrical gadgets, it is also highly useful in the sports and electrical industries. Polythiophene are used to treat prion disorders. It is also capable of detecting metal ions.The development of contemporary plastics was influenced by the creation of Bakelite, which finds use in a variety of sectors including the jewelry, electrical, clocks, kitchenware and sports. The functions of DNA include information encoding, replication, mutation, and recombination gene expression.Generic applications of metric dimension in chemical graph theory is discussed. In future, definitely metric dimension will play an important role in understanding the behaviour of chemical compounds. All the networks discussed in current manifesto have constant Edge metric dimension except Bakelite network.

Our research now raises a few unresolved issues and problems.

Open question 1.: Investigate the edge metric dimension of polythiophene network.
Open question 2.: Investigate the edge metric dimension of Backbone DNA network.

## Data Availability

All data used in the article are directly available in the text.

## Abbreviations

$(CNBH_{(m)})$, $(PT_{(m)})$, $BB_{DNA_{(m)}}$: “m” is the number of units
g,h,i,j,k,l,g′,h′,i′,j′,k′: $$(CNBH_{(m)})$$ Vertex labels
$A_1, A_2$: First and second vertex in $PT_{(m)}$
K,L,M,N,O,P,Q: $$PT_{(m)}$$ Vertex labels
A,B,C,D,E: $$BB_{DNA_{(m)}}$$ Vertex labels
$X_1, X_2$: First and second vertex in $BB_{DNA_{(m)}}$
$(B(n \times m))$: “n,m”= number of rows and columns
*H*: Resolving set in $(B(n\times m))$
